# The impact of factor Xa inhibitors on bleeding risk in patients with respiratory diseases

**DOI:** 10.1038/s41598-024-54714-5

**Published:** 2024-02-19

**Authors:** Shohei Hamada, Kei Muramoto, Kimitaka Akaike, Hiroko Okabayashi, Aiko Masunaga, Yusuke Tomita, Hidenori Ichiyasu, Takuro Sakagami

**Affiliations:** Department of Respiratory Medicine, Kumamoto University Hospital, Faculty of Life Sciences, Kumamoto University, 1-1-1 Honjo, Chuo-ku, Kumamoto 860-8556 Japan

**Keywords:** Outcomes research, Circulation, Respiration

## Abstract

It is unclear which factor Xa (FXa) inhibitors are associated with higher bleeding risk in patients with respiratory diseases, and there are no studies on the association between prothrombin time–international normalized ratio (PT–INR) and bleeding risk. We conducted a retrospective cohort study comparing 1-year-outcomes and PT–INR between patients with respiratory diseases treated with rivaroxaban (R group, n = 82) or edoxaban (E group, n = 138) for atrial fibrillation or venous thromboembolism from 2013 to 2021. The most frequent event of all bleeding discontinuations was respiratory bleeding in both groups (7.3 and 4.3%, respectively). The cumulative incidence of bleeding discontinuation was significantly higher in the R group (25.6%) than in the E group (14.4%) (hazard ratio [HR], 2.29; 95% confidence interval [CI] 1.13–4.64; *P* = 0.023). PT–INR after initiation of therapy significantly increased and was higher in the R group than in the E group (median value, 1.4 and 1.2, respectively; *P* < 0.001). Multivariate analysis using Cox proportional hazards and Fine-Gray models revealed that PT–INR after initiation of therapy was an independent risk factor of bleeding discontinuation events (HR = 4.37, 95% CI 2.57–7.41: *P* < 0.001). Respiratory bleeding occasionally occurs in patients receiving FXa inhibitors, and monitoring the PT–INR may need to ensure safety.

## Introduction

Direct oral anticoagulant agents (DOACs), including thrombin inhibitors (dabigatran) and factor Xa (FXa) inhibitors (apixaban, rivaroxaban, and edoxaban), are recommended as the first choice for anticoagulation in patients with venous thromboembolism (VTE)^1,2^ and nonvalvular atrial fibrillation (AF)^3^. Several randomized controlled trials (RCTs) have shown that the efficacy and safety of DOACs are either equivalent, or superior to those of vitamin K antagonists (VKA), which are the conventional standard treatment drugs^4–12^. The advantages of DOACs compared to VKA are that they are not influenced by diet, are effective soon after administration, have relatively few cross-drug interactions, and cause less intracranial bleeding.

Another advantage of DOACs is that unlike VKA, they do not require routine monitoring to ensure safety^13,14^. Nevertheless, a correlation between blood drug concentration and prothrombin time (PT) or PT-international normalized ratio (PT–INR) is known for the FXa inhibitors, riveroxaban and edoxaban^15–19^. It has also been suggested that bleeding risk may increase as drug concentrations in blood increase^20^, but the association between PT/PT–INR and the occurrence of bleeding events has not been studied.

Disadvantages of DOACs include lack of antagonists, restriction of use in patients with renal dysfunction, and a higher frequency of gastrointestinal bleeding than that with VKA usage^21^. In addition, there is apparently lack of clarity regarding the safety profile for usage of DOACs. The bleeding risk in the real-world settings may differ from that in clinical trials, and the differences in the safety profile of each DOACs and various pre-existing background diseases have not been sufficiently examined. Respiratory diseases, such as interstitial lung disease (ILD), chronic obstructive pulmonary disease (COPD), and lung cancer, are known to have a high incidence of thrombosis^22–24^. However, the characteristics of bleeding events concomitant with DOACs use are still unclear, although these patients sometimes develop hemoptysis or alveolar hemorrhage due to alveolar vasculitis, lung infections, and lung cancer^25–27^.

Therefore, we conducted a single-center retrospective study to examine the characteristics of the bleeding events and compare the bleeding risk for two FXa inhibitor, rivaroxaban or edoxaban, in patients with respiratory diseases. In addition, the association between PT–INR and bleeding risk was analyzed, and predictors of clinically significant bleeding events were identified.

## Results

### Patient characteristics

**Figure 1:**
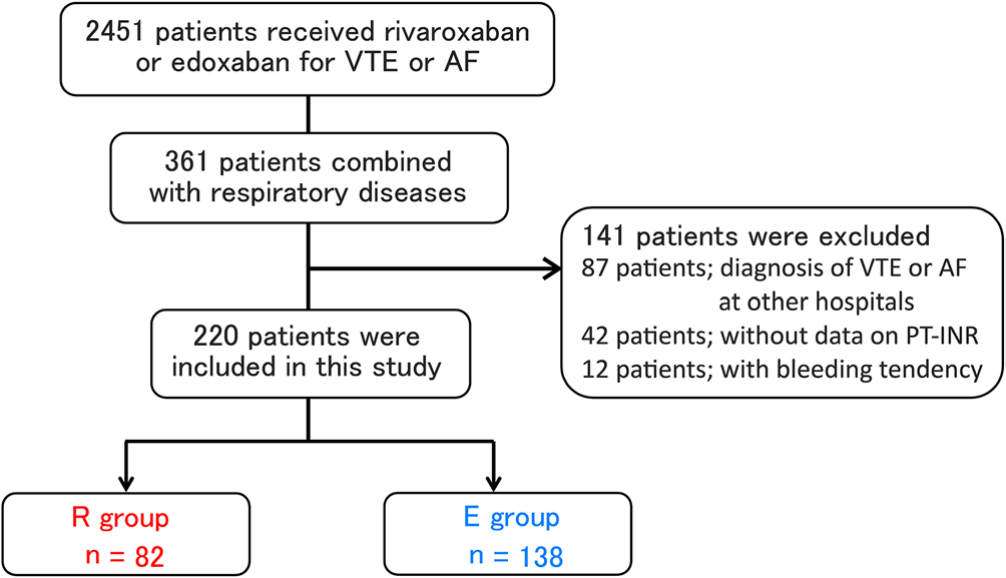
Schematic diagram of patient selection.

Of the total 2451 patients receiving rivaroxaban or edoxaban for VTE or AF during the study period, 361 patients receiving these two FXa inhibitors for concomitant presence of other respiratory diseases were enrolled. From these, 141 patients were excluded owing to diagnosis of VTE or AF at other hospitals (n = 87), lack of data on PT–INR (n = 42), and bleeding tendency (n = 12). A total of 220 patients were included in this study, of which 82 (37%) and 138 (63%) patients were treated with rivaroxaban and edoxaban, respectively (Fig. 1).

The baseline patient characteristics are shown in Table 1. The most common underlying pulmonary disease was lung cancer (34.5%), followed by chronic obstructive pulmonary disease (31.4%), and interstitial lung disease (30.9%). Post thoracic surgery (20.5%) included operations for lung tumors and other pulmonary diseases, pleural diseases, and mediastinal tumors. There were no significant differences between the two groups except for median weight, proportion of AF, deep vein thrombosis (DVT), and use of macrolide antibiotics. The initial therapeutic doses of the FXa inhibitors are shown in Supplementary Table S1. The frequency of the maximum dose of each agent was 35.4% in group R and 26.8% in group E, with no significant difference between the two groups (*P* = 0.224).

**Table 1:** Baseline characteristics of patients.

	All (*n* = 220)	R group (*n* = 82)	E group (*n* = 138)	*P* value
Age (years)	71.0 (66.0–76.0)	70.0 (63.8–74.3)	72.0 (66.8–78.0)	0.080
Male, sex	148 (67.3)	62 (75.6)	86 (62.3)	0.053
Weight (kg)	59.0 (49.8–65.5)	62.0 (52.5–66.6)	55.5 (48.0–64.2)	**0.010**
Ever-Smokers	141 (64.1)	59 (72.0)	82 (59.4)	0.081
AF	84 (38.2)	40 (48.8)	44 (31.9)	0.015
VTE	139 (63.2)	41 (50.0)	98 (71.0)	**0.002**
DVT	126 (57.3)	33 (40.2)	93 (67.4)	** < 0.001**
PTE	44 (20.0)	18 (22.0)	26 (18.8)	0.604
Pulmonary disease				
Lung cancer	76 (34.5)	29 (35.4)	47 (34.1)	0.884
COPD	69 (31.4)	30 (36.6)	39 (28.3)	0.230
ILD	68 (30.9)	26 (31.7)	42 (30.4)	0.881
Bronchial asthma	47 (5.8)	17 (20.7)	30 (21.7)	1.000
Post thoracic surgery	45 (20.5)	22 (26.8)	23 (16.7)	0.084
Bronchiectasis	11 (5.0)	3 (3.7)	8 (5.8)	0.750
NTM	7 (3.2)	2 (2.4)	5 (3.6)	1.000
Others	23 (10.5)	9 (11.1)	14 (10.1)	1.000
Antiplatelet agents	37 (16.8)	11 (13.4)	26 (18.8)	0.354
Interacting drugs with FXa inhibitors	85 (38.6)	26 (31.7)	59 (42.8)	0.117
NSAIDs	40 (11.8)	10 (12.2)	30 (21.7)	0.103
SSRI	26 (11.5)	13 (15.9)	13 (9.4)	0.195
Macrolide antibiotics	17 (7.7)	2 (2.4)	15 (10.9)	**0.027**
Herbesser	7 (3.2)	3 (16.7)	3 (8.8)	0.406
Laboratory findings				
CRP (mg/dL)	0.9 (0.2–4.0)	0.8 (0.2–4.0)	0.9 (0.2–4.3)	0.787
eGFR (mL/min/1.73m^2^)	60.2 (42.3–81.5)	71.0 (62.0–86.0)	64.0 (41.4–73.3)	0.176

Data are presented as median (range) or N (%). *R group* receiving rivaroxaban, *E group* receiving edoxaban, *AF* atrial fibrillation, *VTE* venous thromboembolism, *DVT* deep venous thrombosis, *PTE* pulmonary thromboembolism, *COPD* chronic obstructive pulmonary disease, *ILD* interstitial lung disease, *NTM* non-tuberculous mycobacteria, *FXa* factor Xa, *NSAIDS* non-steroidal anti-inflammatory drugs, *SSRI* selective serotonin reuptake inhibitors, *CRP* C-reactive protein, *eGFR* estimated glomerular filtration rate.

Significant values are in bold.

### The incidence of discontinuation of therapy

**Figure 2:**
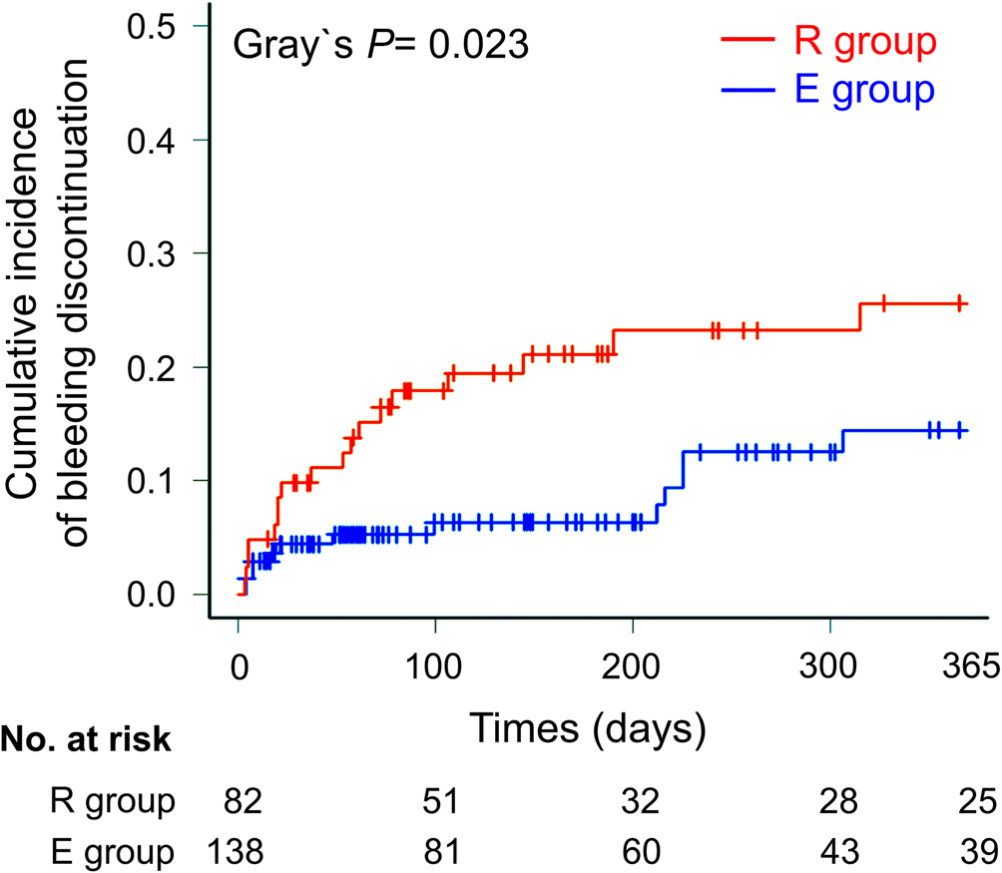
The difference between cumulative incidence curves of bleeding discontinuation during observational period among the patients treated with rivaroxaban or edoxaban. The red and blue lines indicate the patients treated with rivaroxaban and edoxaban, respectively.

All the discontinuation events are listed in Table 2. Major bleeding occurred in 9.8 and 5.1% patients in the R and E groups, respectively (*P* = 0.268). The incidence of bleeding discontinuation was higher than that of competing risk factors that induced discontinuation of therapy, such as other adverse events and thrombosis events in both groups. Of all bleeding discontinuation factors, respiratory bleeding was most frequent in both groups. The cumulative incidence of bleeding discontinuation was 25.6% in the R group and 14.4% in the E group (hazard ratio [HR] with rivaroxaban, 2.26; 95% confidence interval [CI], 1.11–4.59; *P* = 0.023; Gray’s test) (Fig. 2). There were no significant differences in the occurrence of competing risk factors, including other adverse and thrombotic events, between the two groups (Supplementary Fig. S1). In patients with VTE and AF, the cumulative incidence of bleeding discontinuation showed no significant difference. (HR with VTE, 0.66; 95% CI 0.33–1.31; *P* = 0.234, Gray test) (Supplementary Fig. S2). In the next subgroup analysis, there was not significantly difference in the risk of bleeding discontinuation between the R group and the E groups in patients with lung cancer (HR with rivaroxaban, 1.33; 95% CI 0.31–5.75; *P* = 0.700, Gray test) and with ILD (HR with rivaroxaban, 2.04; 95% CI 0.68–6.14; *P* = 0.200, Gray test). (Supplementary Table. S2).

**Table 2:** Clinical outcomes during study period.

	All (n = 220)	R group (n = 82)	E group (n = 138)	*P* value
Observational period, days (median)	152 (56–365)	153 (58–365)	149 (51–365)	0.536
Major bleeding	15 (6.8)	8 (9.8)	7 (5.1)	0.268
Respiratory bleeding	7 (3.2)	3 (3.7)	4 (2.8)	0.714
Gastrointestinal bleeding	7 (3.2)	4 (4.9)	3 (2.2)	0.429
Bloody urine	1 (0.5)	1 (1.2)	0 (0)	0.373
Discontinuation events of FXa inhibitors therapy	50 (22.7)	24 (29.2)	26 (18.8)	0.096
Bleeding discontinuation	31 (14.1)	18 (22.0)	13 (9.4)	**0.015**
Respiratory bleeding	12 (5.5)	6 (7.3)	6 (4.3)	0.370
Gastrointestinal bleeding	10 (4.5)	5 (6.1)	5 (3.6)	0.506
Nose bleeding	4 (1.8)	3 (3.7)	1 (1.4)	0.147
Subcutaneous bleeding	2 (0.9)	2 (2.4)	0 (0)	0.138
Bloody urine	2 (0.9)	1 (1.2)	1 (0.7)	1.000
Genital bleeding	1 (0.5)	1 (1.2)	0 (0)	0.373
Adverse events other than bleeding	11 (5.0)	4 (4.9)	7 (5.1)	1.000
Renal disorder	4 (1.8)	1 (1.2)	3 (2.2)	1.000
Digestive symptoms	3 (1.4)	1 (1.2)	2 (1.4)	1.000
Others	4 (1.8)	2 (2.4)	2 (1.4)	0.630
Thrombosis events	8 (3.6)	2 (2.4)	6 (4.3)	1.000
Cerebral infarction	4 (1.8)	0 (0)	4 (2.8)	0.299
Recurrence or development of VTE	4 (1.8)	2 (2.4)	2 (1.4)	0.630

Data are presented as median (range) or N (%). *R group* receiving rivaroxaban, *E group* receiving edoxaban, *FXa* factor Xa, *VTE* venous thromboembolism.

Significant values are in bold.

### The changes of PT–INR

A comparison of the PT–INR between the two groups is shown in Table 3. The PT–INR before initiation of FXa inhibitor therapy, and at the time of bleeding discontinuation were not significantly different between the two groups. In contrast, PT–INR after initiation of therapy was higher in the R group than in the E group (median value, 1.4 vs. 1.2; *P* < 0.001). In both groups, PT–INR significantly increased before and after treatment initiation (R group: *P* < 0.001, E group: *P* < 0.001; Supplementary Fig. S3). Linear regression analysis for PT–INR after initiation of therapy was performed. Multivariable analysis showed that rivaroxaban use (regression coefficient [B] = 0.18 standard error [SE:0.04], β = 0.35, *P* < 0.001), and PT–INR before the initiation of therapy (B = 0.67 [0.11], β = 0.35, *P* < 0.001) were significant factors (Supplementary Table S3).

**Table 3:** The changes in PT–INR with FXa inhibitor therapy.

	R group HR (95% CI) (n = 82)	E group HR (95% CI) (n = 138)	*P* value
PT–INR before initiation of therapy	1.1 (1.0–1.1)	1.0 (1.0–1.1)	0.191
PT–INR after initiation of therapy	1.4 (1.1–1.9)	1.2 (1.0–1.3)	** < 0.001**
PT–INR at bleeding discontinuation	2.3 (2.0–2.5)	2.0 (1.6–2.2)	0.133
Number of PT–INR measurement days between begore and after initiation of therapy	4.0 (3.0–20.0)	4.0 (2.0–15.0)	0.381

Data are presented as HR (95% CI). *R group* receiving rivaroxaban, *E group* receiving edoxaban; *FXa* factor Xa; *HR* hazard ratio; *CI* confidence interval, *PT–INR* prothrombin time international normalized ratio.

Significant values are in bold.

### Predictive factors of bleeding discontinuation

**Figure 3:**
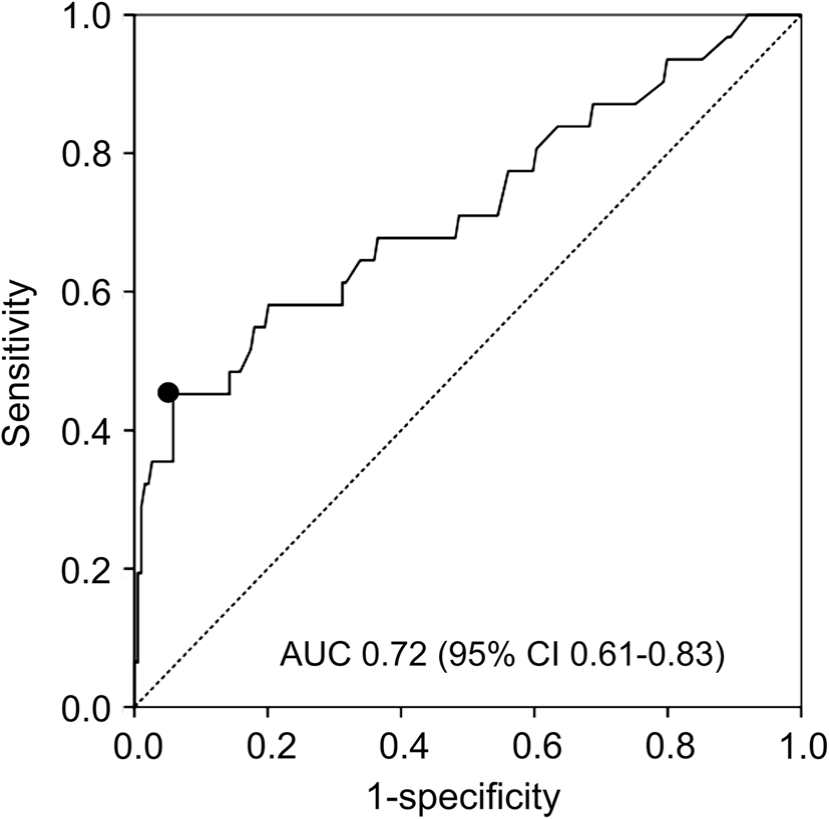
ROC analysis of discrimination ability of the optimal cut-off values of PT–INR after initiation of therapy to discriminate bleeding discontinuation. PT–INR, prothrombin time international normalized ratio.

The results of the Cox proportional hazards analysis with the Fine-Gray model for aborted bleeding discontinuation are shown in Table 4. In univariate analysis, rivaroxaban administration, PT–INR after initiation of therapy, and use of non-steroidal anti-inflammatory drugs (NSAIDs) were identified as significant factors. Multivariate analysis using these factors showed that PT–INR after initiation of therapy (HR = 4.37, 95% CI 2.57–7.41: *P* < 0.001) and NSAIDs use (HR = 2.67, 95% CI 1.28–5.55: *P* = 0.009) were independent predictors. The discriminant ability of PT–INR values and optimal cut-off values for ROC analysis are shown in Fig. 3. The Area under the ROC Curve (AUC) was 0.72 (95%CI: 0.61–0.83). The optimal cut-off value for the PT–INR after initiation of therapy was 1.79 and the sensitivity and specificity were 0.45 and 0.94, respectively.

**Table 4:** Multivariate analyses of predictors of bleeding discontinuation.

Variables	HR (95% CI)	*P* value
Univariate analysis		
Rivaroxaban administration	2.35 (1.16–4.77)	**0.018**
NSAIDs use	2.64 (1.23–5.64)	**0.012**
PT–INR after initiation of therapy	4.54 (2.88–7.16)	** < 0.001**
Multivariate analysis		
NSAIDs use	2.67 (1.28–5.55)	**0.009**
PT–INR after initiation of therapy	4.37 (2.57–7.41)	** < 0.001**

Data are presented as HR (95% CI). *R group* receiving rivaroxaban, *E group*: receiving edoxaban, *HR* hazard ratio, *CI* confidence interval, *NSAIDs* non-steroidal anti-inflammatory drugs, *PT–INR* prothrombin time international normalized ratio.

Significant values are in bold.

## Discussion

We examined the impact of FXa inhibitors on the PT–INR and risk of bleeding in patients with respiratory diseases. Rivaroxaban was found to be associated with increased PT–INR more than edoxaban did, and the cumulative incidence of bleeding discontinuation was higher in the R group than in the E group. Multivariable analysis demonstrated that after initiation of the therapy, PT–INR and NSAIDs use were independent risk factors for bleeding discontinuation.

Few studies have investigated the efficacy and safety of DOACs for various pre-existing background disease. According to the Hokusai VTE Cancer Clinical Trial and the SELECT-D trial^29,30^, the bleeding risk was higher with rivaroxaban and edoxaban than with dalteparin in patients with various cancers. One of the strengths of this study is that, this is the first time safety profiles of FXa inhibitors in patients with respiratory diseases has been examined, with revelation of high frequency of respiratory bleeding in such cases. It is particularly important to identify the optimal anticoagulation therapy for this population, because patients with lung cancer, COPD, and ILD commonly develop VTE as a complication, often concomitant with cardiac disease, including AF^22–24^. The progression of lung cancer can induce bloody sputum or hemoptysis; and COPD and ILD are often accompanied by bronchopulmonary structural destruction and angiogenesis, which can lead to respiratory bleeding due to pulmonary infection, pulmonary edema, and vasculitis^25–27^. Thus, the incidence of respiratory bleeding was the marked type among all bleeding discontinuations and major bleeding events in this study. It should also be noted that this population often uses drugs which can cross-react with FXa inhibitors, such as NSAIDs for cancer pain and macrolide antibiotics for chronic respiratory infections, including *Pseudomonas aeruginosa* or non-tuberculosis mycobacteria treatment. Although macrolide antibiotics were not a predictor for either bleeding discontinuations or PT–INR after initiation of treatment in multivariate analysis, NSAIDs use was an independent risk factor for bleeding discontinuation in the present study.

Based on the results of several previous randomized controlled trials (RCTs) in patients with VTE and AF, treatment with DOACs is perceived to be as effective as with VKA and are associated with decrease in severity and frequency of bleeding^4–12^. However, RCTs are conducted under idealized and rigorously controlled conditions. The real-world clinical outcomes of DOAC-treated patients are not necessarily consistent with those of RCTs. The number of incidence of major bleeding in patients with VTE or AF has been reported to be 1.1–3.6%/year in RCTs^6–11^, 5.0–5.7%/year in real-world studies^31–33^, and 6.8%/year in our study. In addition, no head-to-head RCTs have compared the safety of different DOACs among themselves. In a network meta-analysis including phase II or phase III RCTs that evaluated the use of DOACs and VKA for AF^34^, usage of edoxaban and rivaroxaban showed a significantly increased risk of major bleeding than apixaban. In an exploratory retrospective cohort study among patients with VTE, the incidence of major bleeding was found to be 4.4%/year in the apixaban group and 5.0%/year in the rivaroxaban group, with an HR of apixaban to be 0.86 (95% CI 0.71 to 1.04)^33^. The present study is the first to compare the bleeding risk in patients with respiratory disease treated with rivaroxaban versus edoxaban, for VTE or AF. Although the incidence of major bleeding was not significantly different, the risk of bleeding discontinuation was significantly higher in the R group than that in the E group. In subgroup analysis of lung cancer and ILD patients, no significant difference in the risk of bleeding discontinuation was found between the R group and the E groups, suggesting that different underlying pulmonary diseases have different impact on bleeding with each FXa inhibitor.

This study is also the first to show PT–INR after initiation of therapy is a predictor of bleeding events in patients with pulmonary diseases. An important aspect of this study is that an association between PT–INR and bleeding risk was demonstrated in the enrolled population. FXa inhibitors have predictable pharmacodynamics, pharmacokinetics, and wide therapeutic windows, and are not inferior in safety compared to VKA. Therefore, routine therapeutic monitoring is not considered necessary^13,14^. However, it has been shown that FXa inhibitors cause more gastrointestinal bleeding than VKA^21^. Thus, a suitable indicator for monitoring the progress is desirable. FXa inhibitors suppress the rate of thrombin production and the amount of thrombin by inhibiting FXa in the prothrombinase complex; this effect should be reflected relatively well in PT values. In fact, some PT assays have been reported to correlate well with the concentrations of rivaroxaban and edoxaban^15–17^. The ISTH guidelines favor PT and the anti-FXa assay as the tests of choice, respectively for screening and quantification of rivaroxaban and apixaban^28^. As the reagents used for performing the PT test may vary among different laboratories, and even within the same laboratory over time, PT–INR was used in this study^18^. Our results showed an increase in PT–INR after initiation of FXa inhibitor therapy compared to before treatment, as well as an increase in PT–INR at the time of bleeding discontinuation. In particular, rivaroxaban administration was an independent risk factor for elevated PT–INR after initiation of therapy. In contrast, multivariable analysis showed that PT–INR after initiation of therapy, but not rivaroxaban administration, was an independent predictor of bleeding discontinuation. This means that rivaroxaban affects PT–INR after the initiation of therapy more than edoxaban does, which leads to higher risk of bleeding in the R group than in the E group. Furthermore, from the ROC curve, the optimal cut-off value of PT–INR after the initiation of therapy to discriminate bleeding discontinuation was 1.79. This preliminary study indicated that strong evidence for monitoring PT–INR value to reduce bleeding risk should be establish by further studies.

This study has several limitations. First, this was a small, retrospective cohort, non-randomized study, which might allow for selection bias, high dropout rate and insufficient sample size to support the research results. The median weight, and ratio of AF, VTE, and the use of macrolide antibiotics were significantly different between the R and E groups although these factors were not associated with discontinuation of bleeding. Furthermore, in order to exclude all confounding factors, a clinical trial with the RCT design was required. Second, the number of days and time of PT–INR measurements after the initiation of therapy were not standardized. This limitation might be particularly related to the low sensitivity of the optimal cut-off value of PT–INR after initiation of treatment to discriminate bleeding discontinuation; this needs be verified in a prospective clinical trial. Third, the different condition of each lung disease may have a impact on the frequency of respiratory bleeding. Finally, the decision to discontinue FXa therapy was made by individual attending physician without any common guidelines.

Our study indicated that FXa inhibitors can occasionally induce respiratory bleeding in patients with respiratory diseases. Although the bleeding risk with rivaroxaban therapy was higher than that with edoxaban, PT–INR after initiation of therapy was a predictor of bleeding discontinuation. To ensure safety, PT–INR should be monitored when administering FXa inhibitors to these patients.

## Methods

### Patient selection

This was a retrospective cohort study. We reviewed the medical records of patients with a history of FXa inhibitor prescription (rivaroxaban or edoxaban), for VTE or AF, in our hospital from April 2013 to April 2021. All patients diagnosed with respiratory disease by respiratory medicine specialists at our hospital were screened for eligibility. Patients were excluded if they had an obvious bleeding tendency (e.g., disseminated intravascular coagulation in critically ill patients, hematologic diseases causing platelet decline, extensive cerebral infarction with a high risk of hemorrhagic infarction), a history of clinically relevant bleeding and a creatinine clearance of < 15 mL/min. Patients who had started FXa inhibitor therapy at another hospital, or those without PT–INR test results were also excluded. The enrolled patients were divided into two groups (R group or E group) according to whether they received rivaroxaban or edoxaban, respectively. This study was conducted in accordance with the principles of the Declaration of Helsinki. The institutional review board of Kumamoto University Hospital approved this study (approval number: 2635), and waived written informed consent because of the retrospective nature of the study design, but we applied Opt-out method and obtained informed consent on the web-site.

### Definition of bleeding events

Bleeding discontinuation was defined as the discontinuation of FXa inhibitor therapy due to adverse bleeding events. Respiratory bleeding included bloody sputum, hemoptysis, and alveolar hemorrhage. In accordance with the criteria of the International Society on Thrombosis and Haemostasis (ISTH), major bleeding was defined as overt bleeding that was associated with a decrease in the hemoglobin level ≥ 2 g/dL, leading to a transfusion of two or more units of blood, occurred at a critical site, or contributed to death^28^

### Outcomes

Enrolled patients were followed-up in our hospital inpatient or outpatient settings. The observation period was defined as the time from the day of initiation of FXa inhibitor therapy (day 0) to day 365, or from day 0 to the day of discontinuation of therapy due to adverse events, including bleeding discontinuation or thrombotic events such as VTE and cerebral infarction. Patients withdrawn from the study due to other causes such as hospital transfer or discontinuation of therapy by patient's request, were censored. The cumulative incidence of bleeding discontinuation during the observation period was compared between Groups R and E. We examined the PT–INR before initiation of FXa inhibitor therapy (before initiation of therapy), at the first measurement within one month after initiation of therapy (after initiation of therapy), and at the time of bleeding discontinuation. PT–INR values before and after the initiation of therapy were compared between the R and E groups. Furthermore, relationship between the PT–INR and bleeding risk was analyzed. Predictive factors for bleeding discontinuation were identified. Patients withdrawn from the study due to cessation of follow-up for reasons other than adverse and thrombotic events, such as hospital transfer, were censored.

### Data collection and statistical analysis

All clinical and laboratory data were collected from the patients’ medical records. Nonparametric summaries were performed for the continuous scale, and the medians and interquartile ranges (IQR) were calculated. The Mann–Whitney U test was used to compare the two groups. The change in the PT–INR with FXa inhibitor therapy was analyzed using the Wilcoxon signed-rank sum test. We conducted survival analysis with bleeding discontinuation as an event. Cumulative incidence analysis (Gray’s test) and Cox proportional hazards model analysis with the Fine-Gray model were performed, in which discontinuation of therapy due to adverse events other than bleeding or thrombosis events were treated as competing risks to bleeding discontinuation. After univariate analysis including all of variables, multivariable analysis was conducted, in which the factors that were significant in the univariate analysis were analyzed using a stepwise variable selection method. Survival Receiver Operating Characteristic (ROC) analysis was performed to evaluate the discriminatory ability of PT–INR for the occurrence of aborted bleeding events, and to calculate the optimal cut-off value. The optimal cutoff value was determined using the Youden Index method. In addition, the effects of patient’s background and drug administration history on PT–INR after therapy initiation were evaluated using linear regression analysis. Multivariable analysis was performed, together with the Cox regression model described above.

A two-sided *P* value of < 0.05 was considered statistically significant. All statistical analyses were performed using SPSS for Windows version 23.0 (IBM Japan, Tokyo, Japan).

### Supplementary Information

Supplementary Information.

## Data Availability

The datasets used or analyzed during the current study available from the corresponding author on reasonable request.

## References

[1] Stevens SM, Woller SC, Kreuziger LB, Bounameaux H, Doerschug K, Geersing GJ (2021). Antithrombotic therapy for VTE disease: Second update of the CHEST guideline and expert panel report. Chest.

[2] Ortel TL, Neumann I, Ageno W, Beyth R, Clark NP, Cuker A (2020). American Society of Hematology 2020 guidelines for management of venous thromboembolism: Treatment of deep vein thrombosis and pulmonary embolism. Blood Adv..

[3] Hindricks G, Potpara T, Dagres N, Arbelo E, Bax JJ, Blomström-Lundqvist C (2021). 2020 ESC Guidelines for the diagnosis and management of atrial fibrillation developed in collaboration with the European Association for Cardio-Thoracic Surgery (EACTS): The Task Force for the diagnosis and management of atrial fibrillation of the European Society of Cardiology (ESC) Developed with the special contribution of the European Heart Rhythm Association (EHRA) of the ESC. Eur. Heart J..

[4] Schulman S, Kakkar AK, Goldhaber SZ, Schellong S, Eriksson H, Mismetti P (2014). Treatment of acute venous thromboembolism with dabigatran or warfarin and pooled analysis. Circulation.

[5] Agnelli G, Buller HR, Cohen A, Curto M, Gallus AS, Johnson M (2013). Oral apixaban for the treatment of acute venous thromboembolism. N. Engl. J. Med..

[6] Buller HR, Decousus H, Grosso MA, Mercuri M, Middeldorp S, Prins MH (2013). Edoxaban versus warfarin for the treatment of symptomatic venous thromboembolism. N. Engl. J. Med..

[7] Buller HR, Prins MH, Lensin AW, Decousus H, Jacobson BF, Minar E (2012). Oral rivaroxaban for the treatment of symptomatic pulmonary embolism. N. Engl. J. Med..

[8] Connolly SJ, Michael D, Yusuf S, Eikelboom J, Oldgren J, Parekh A (2009). Dabigatran versus warfarin in patients with atrial fibrillation. N. Engl. J. Med..

[9] Granger CB, Alexander JH, McMurray JJV, Lopes RD, Hylek EM, Hanna M (2011). Apixaban versus warfarin in patients with atrial fibrillation. N. Engl. J. Med..

[10] Giugliano RP, Ruff CT, Braunwald E, Murphy SA, Wiviott SD, Halperin JL (2013). Edoxaban versus warfarin in patients with atrial fibrillation. N. Engl. J. Med..

[11] Patel MR, Mahaffey KW, Garg J, Pan G, Singer DE, Hacke W (2011). Rivaroxaban versus warfarin in nonvalvular atrial fibrillation. N. Engl. J. Med..

[12] Dogliotti A, Paolasso E, Giugliano RP (2014). Current and new oral antithrombotics in non-valvular atrial fibrillation: A network meta-analysis of 79808 patients. Heart.

[13] Trujillo T, Dobesh P (2014). Clinical use of rivaroxaban: Pharmacokinetic and pharmacodynamic rationale for dosing regimens in different indications. Drugs.

[14] Bounameaux H, Reber G (2010). New oral anticoagulants: A need for laboratory monitoring. Against. J. Thromb. Haemost..

[15] Samuelson BT, Cuker A, Siegal DM, Crowther DM, Garcia DA (2017). Laboratory assessment of the anticoagulant activity of direct oral anticoagulants. A systematic review. CHEST.

[16] Gosselin RC, Adcock D, Hawes EM, Francart SJ, Grant RP, Mollet S (2015). Evaluating the use of commercial drug-specific calibrators for determining PT and APTT reagent sensitivity to dabigatran and rivaroxaban. Thromb. Haemost..

[17] Van Blerk M, Bailleul E, Chatelain B, Demulder A, Devreese K, Douxfils J (2015). Influence of dabigatran and rivaroxaban on routine coagulation assays: A nationwide Belgian survey. Thromb. Haemost..

[18] Tripodi A, Chantarangkul V, Guinet C, Samamaet MM (2011). The international normalized ratio calibrated for rivaroxaban has the potential to normalize prothrombin time results for rivaroxaban-treated patients: Results of an in vitro study. J. Thromb. Haemost..

[19] Mendell J, Tachibana M, Shi M, Kunitada S (2011). Effects of food on the pharmacokinetics of edoxaban, an oral direct factor Xa inhibitor, in healthy volunteers. J. Clin. Pharmacol..

[20] Sennesael A, Larock A, Douxfils J, Elens L, Stillemans G, Wiesen M (2018). Rivaroxaban plasma levels in patients admitted for bleeding events: Insights from a prospective study. Thromb. J..

[21] Ruff CT, Giugliano RP, Braunwald E, Hoffman EB, Deenadayalu N, Ezekowitz MD (2014). Comparison of the efficacy and safety of new oral anticoagulants with warfarin in patients with atrial fi brillation: A meta-analysis of randomised trials. Lancet.

[22] Pastori D, Marang A, Bisson A, Menichelli D, Herbert J, Lipet GYH (2021). Thromboembolism, mortality, and bleeding in 2,435,541 atrial fibrillation patients with and without cancer: A nationwide cohort study. Cancer.

[23] Sprunger DB, Olson AL, Huie TJ, Fernandez-Perez ER, Fischer A, Solomon JJ (2012). Pulmonary fibrosis is associated with an elevated risk of thromboembolic disease. Eur. Respir. J..

[24] Kubota Y, London SJ, Cushman M, Chamberlain AM, Rosamond WD, Heckbertet SR (2016). Lung function, respiratory symptoms and venous thromboembolism risk: The Atherosclerosis Risk in Communities Study. J. Thromb. Haemost..

[25] Horsted F, West J, Grainge MJ (2012). Risk of venous thromboembolism in patients with cancer: A systematic review and meta-analysis. PLoS Med..

[26] Earwood JS, Thompson TD (2015). Hemoptysis: Evaluation and management. Am. Fam. Physician.

[27] Lara AR, Schwarz MI (2010). Diffuse alveolar hemorrhage. CHEST.

[28] Baglin T, Hillarp A, Tripodi A, Elalamy I, Buller H, Agenoet W (2013). Measuring Oral Direct Inhibitors (ODIs) of thrombin and factor Xa: A recommendation from the Subcommittee on Control of Anticoagulation of the Scientific and Standardisation Committee of the International Society on Thrombosis and Haemostasis. J. Thromb. Haemost..

[29] Raskob GE, Nick VE, Verhamme P, Carrier PM, Nisio MD, Garcia D (2018). Edoxaban for the treatment of cancer-associated venous thromboembolism. N. Engl. J. Med..

[30] Young AM, Marshall A, Thirlwall J, Chapman O, Lokare A, Hill C (2018). Comparison of an oral factor Xa inhibitor with low molecular weight heparin in patients with cancer with venous thromboembolism: Results of a randomized trial (SELECT-D). J. Clin. Oncol..

[31] Gregory YHL, Keshishian A, Li X, Hamilton M, Masseria C, Gupta K (2018). Effectiveness and safety of oral anticoagulants among nonvalvular atrial fibrillation patients. Stroke.

[32] Köhler C, Tittl L, Marten S, Naue C, Spindler M, Stannek L (2022). Effectiveness and safety of edoxaban therapy in daily-care patients with atrial fibrillation. Results from the DRESDEN NOAC REGISTRY. Thromb. Res..

[33] Pawar A, Gagne JJ, Gopalakrishnan C, Iyer G, Tesfaye H, Brill G (2022). Association of type of oral anticoagulant dispensed with adverse clinical outcomes in patients extending anticoagulation therapy beyond 90 days after hospitalization for venous thromboembolism. JAMA.

[34] López-López JA, Sterne JAC, Thom HHZ, Higgins JPT, Hingorani AD, Okoliet GN (2017). Oral anticoagulants for prevention of stroke in atrial fibrillation: Systematic review, network meta-analysis, and cost effectiveness analysis. BMJ.

