# Supplementary material for: The impact of factor Xa inhibitors on bleeding risk in patients with respiratory diseases
Source: Sci Rep. 2024 Feb 19;14:4039. doi: 10.1038/s41598-024-54714-5 (PMC10874933; doi:10.1038/s41598-024-54714-5)
Supplement: Supplementary file 1 — Supplementary Information. [file 41598_2024_54714_MOESM1_ESM.pdf]

Supplementary Information

Impact of factor Xa inhibitors on bleeding risk in patients with respiratory diseases

Shohei Hamada*, Kei Muramoto, Kimitaka Akaike, Hiroko Okabayashi, Aiko Masunaga, Yusuke Tomita, Hidenori Ichiyasu, Takuro Sakagami

Supplementary Table S1. Therapeutic doses of rivaroxaban and edoxaban

Dose, N (%)	Rivaroxaban N (%)	Edoxaban N (%)
10 mg/day	11 (13.4)	-
15 mg/day	42 (51.2)	4 (2.9)
30 mg/day	29 (35.4)	97 (70.3)
60 mg/day	-	37 (26.8)

Supplementary Table S2. Subgroup analysis of patients with lung cancer and ILD comparing the risk of bleeding discontinuation between the R and E groups

	**HR with rivaroxaban (95% CI)**	***P* value**
**Subgroup**		
Patients with lung cancer	1.33 (0.31–5.75)	0.700
Patients without lung cancer	2.78 (1.23–6.31)	0.014
Patients with ILD	2.04 (0.68–6.14)	0.200
Patients without ILD	2.04 (1.01–6.11)	0.048

Supplementary Table S3. Multivariate linear regression analysis to predict PT-INR after initiation of therapy

Variables	B	SE	β	*P* value
**Univariate analysis**				
ILD	0.077	0.037	0.139	**0.039**
VTE	-0.074	0.036	-0.139	**0.039**
Rivaroxaban administration	0.187	0.034	0.354	**<0.001**
PT-INR before initiation of therapy	0.755	0.118	0.398	**<0.001**
**Multivariate analysis**				
Rivaroxaban administration	0.166	0.031	0.313	**<0.001**
PT-INR before initiation of therapy	0.689	0.112	0.363	**<0.001**

B: regression coefficient; SE: standard error; ILD, interstitial lung disease; VTE, venous thromboembolism; PT-INR, prothrombin time international normalized ratio.

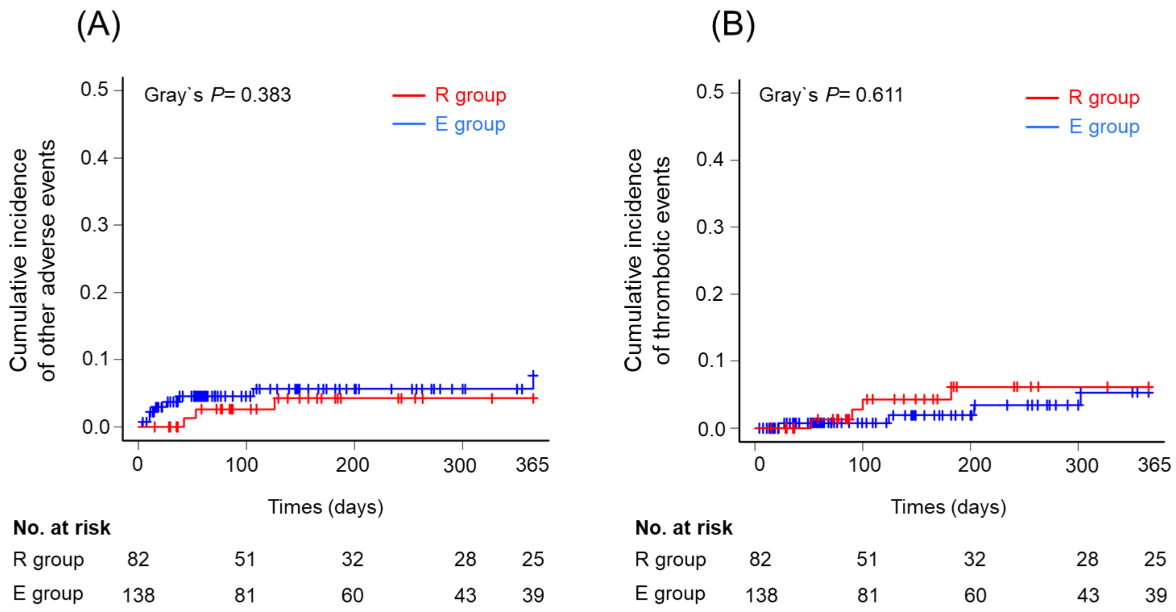

Supplementary Figure S1. The difference of cumulative incidence curves of competing risk events during the observational period. (A) adverse events other than bleeding and (B) thrombotic events. The red and blue lines indicate the patients treated with rivaroxaban or edoxaban, respectively.

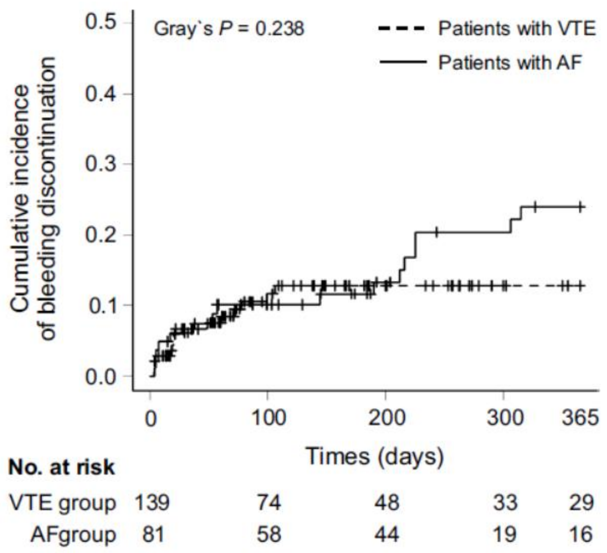

Supplementary Figure S2. The difference of cumulative incidence curves of bleeding discontinuation during observational period between patients with AF or VTE. The solid and dotted lines indicate the patients with AF or VTE, respectively. AF, atrial fibrillation; VTE, venous thromboembolism. AF: atrial fibrillation; VTE: venous thromboembolism.

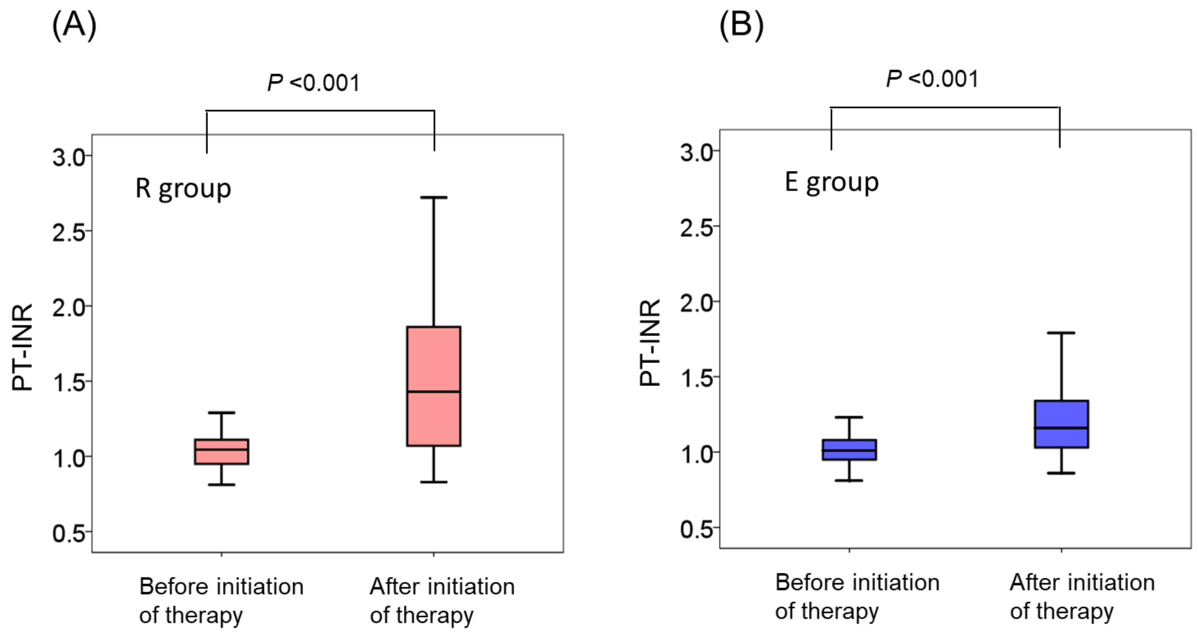

Supplementary Figure S3. The change in PT-INR before and after initiation of FXa therapy to. (A) rivaroxaban group and (B) edoxaban group. PT-INR: prothrombin time international normalized ratio; factor Xa: FXa.
